# Identification of genetic interaction networks via an evolutionary algorithm evolved Bayesian network

**DOI:** 10.1186/s13040-016-0094-4

**Published:** 2016-05-10

**Authors:** Ruowang Li, Scott M. Dudek, Dokyoon Kim, Molly A. Hall, Yuki Bradford, Peggy L. Peissig, Murray H. Brilliant, James G. Linneman, Catherine A. McCarty, Le Bao, Marylyn D. Ritchie

**Affiliations:** Center for Systems Genomics, Department of Biochemistry and Molecular Biology, Pennsylvania State University, University Park, Pennsylvania, USA; Biomedical Informatics Research Center, Marshfield Clinic Research Foundation, Marshfield, Wisconsin USA; Essentia Rural Health, Duluth, Minnesota USA; Department of Statistics, Pennsylvania State University, University Park, Pennsylvania, USA; Biomedical & Translational Informatics, Geisinger Health System, Danville, Pennsylvania USA

**Keywords:** Evolution algorithm, Bayesian Network, Genetic interactions, Discriminant analysis, Type 2 diabetes

## Abstract

**Background:**

The future of medicine is moving towards the phase of precision medicine, with the goal to prevent and treat diseases by taking inter-individual variability into account. A large part of the variability lies in our genetic makeup. With the fast paced improvement of high-throughput methods for genome sequencing, a tremendous amount of genetics data have already been generated. The next hurdle for precision medicine is to have sufficient computational tools for analyzing large sets of data. Genome-Wide Association Studies (GWAS) have been the primary method to assess the relationship between single nucleotide polymorphisms (SNPs) and disease traits. While GWAS is sufficient in finding individual SNPs with strong main effects, it does not capture potential interactions among multiple SNPs. In many traits, a large proportion of variation remain unexplained by using main effects alone, leaving the door open for exploring the role of genetic interactions. However, identifying genetic interactions in large-scale genomics data poses a challenge even for modern computing.

**Results:**

For this study, we present a new algorithm, Grammatical Evolution Bayesian Network (GEBN) that utilizes Bayesian Networks to identify interactions in the data, and at the same time, uses an evolutionary algorithm to reduce the computational cost associated with network optimization. GEBN excelled in simulation studies where the data contained main effects and interaction effects. We also applied GEBN to a Type 2 diabetes (T2D) dataset obtained from the Marshfield Personalized Medicine Research Project (PMRP). We were able to identify genetic interactions for T2D cases and controls and use information from those interactions to classify T2D samples. We obtained an average testing area under the curve (AUC) of 86.8 %. We also identified several interacting genes such as *INADL* and *LPP* that are known to be associated with T2D.

**Conclusions:**

Developing the computational tools to explore genetic associations beyond main effects remains a critically important challenge in human genetics. Methods, such as GEBN, demonstrate the utility of considering genetic interactions, as they likely explain some of the missing heritability.

## Background

Over the past decade, development in large-scale, high-throughput methods to characterize the human genome has dramatically improved our ability to assess the relationship between an individuals’ genome and diseases [1]. With the ever-increasing generation of genomic data, development of computational methods necessary to analyze the vast amount of data are becoming increasingly important [2]. The genome-wide association study (GWAS) was the pioneering method to interrogate the genotypic and phenotypic relationship and is still being widely used today [3, 4]. However, despite GWAS’ wide success in finding associated SNPs in many common diseases, it lacks the power to detect more complex genetic architectures such as genetic interactions [5]. Therefore, a more comprehensive analysis method that can detect both main effects as well as genetic interactions is needed.

Much variability in human diseases and traits remain unexplained by using GWAS alone [5]. It is hypothesized that some of the missing variability could stem from complex genetic interactions that are unexplored by traditional association analysis. Furthermore, studies that do explore genetic interactions are often limited to two-way interactions due to the exponential increase of computational burden associated with higher-way interactions [6]. A number of analytic methods have been proposed and implemented to explore interactions using statistical and data mining strategies. For example, MDR [7, 8] can exhaustively evaluate all possible n-way interactions for a given n and selects the best model based on cross validations. Network based methods such as Neural Networks [9, 10] and Bayesian Networks [11] use their respective network structures to model interactions. Other notably machine learning methods including random forest [12] and SURF [13] use variable importance score to select potential interacting variables that are predictive of the outcome. However, strategies that employ exhaustive search are difficult to scale up due to the exponentially increasing search space. Machine learning methods are more flexible but they often suffer in model interpretability. Typically, the underlying pattern in data is not known a priori, thus it is important to develop a flexible method to model different types of genetic architecture.

To capture main effects of genetic variants as well as complex genetic interactions, we created the Grammatical Evolution Bayesian Network (GEBN) algorithm. The algorithm can simultaneously identify marginal effects as well as interaction effects without exponentially increasing the search time. GEBN can also identify interactions that occur between different sets of genetic variants in different groups (i.e. cases and controls). This flexibility allows discovery of non-overlapping genetic architectures in multiple groups. Previous Bayesian Networks methods to detect genetic interactions [11, 14] have been limited to a small set of input SNPs. Here, we specifically chose to implement an evolutionary computation strategy to evolve the structure of the Bayesian Network because it allows us to model a larger number of SNPs while controlling for the computational time.

We implemented GEBN algorithm in the software package ATHENA. We tested the algorithm on various simulation datasets. We also applied GEBN to a case–control dataset for type 2 diabetes obtained from the Marshfield Personalized Medicine Research Project Biobank (Marshfield PMRP) [15]. The network models identified novel interaction networks for type 2 diabetes cases and healthy individuals, respectively. Using the interaction networks for the two groups, we built prediction models that have an average AUC of 86 %. In the following sections, we describe the GEBN algorithm, data simulations and the application in type 2 diabetes. Our results demonstrate the promise of methods like GEBN.

## Methods

### Grammatical Evolution Bayesian Network (GEBN)

Bayesian Network is a multivariate modeling method that expresses the relationship of variables through a series of conditional distributions. The use of Bayesian Networks is becoming very important in biology because of their ability to infer biological networks [16], model signaling pathways [17], and classifications [18, 19]. The current obstacle for the application of Bayesian Networks in large-scale genomics data is the exponentially increase of search space with the increase of input variables. Thus, we used a grammatical evolution (GE) algorithm to evolve Bayesian Networks in order to reduce computational time. GE is a type of genetic programming [20, 21] that uses Backus-Naur Form (BNF) grammar to create a model based on a genetic algorithm. The advantage of GE algorithm lies in its guided random search so that the search space is greatly reduced. The steps of the GE algorithm is the following:

1. Divide the data into five equal parts for cross-validations
  For each cross validation:
2. Populations of binary string are randomly generated and translated into functional Bayesian Networks by the grammar. For each individual genome, the binary string is divided into consecutive codons. The codons are then translated according to the grammar (Fig. 1).

**Fig. 1:**
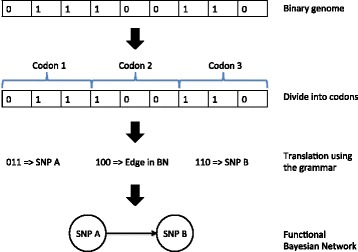
Generation of BN using the grammar
3. Calculate the fitness of the Bayesian Networks using the K2 scoring function [22].
  $$ P\left({B}_s,D\right)=P\left({B}_s\right)\prod_{i=l}^n\prod_{j-1}^{q_i}\frac{r_i=1!}{\left({N}_{ij}+{r}_i-1\right)}{N}_{ij}\prod_{k=1}^{r_i}{N}_{ijk}! $$
  Where D is the dataset, B is Bayesian Network, n is total number of variables, q_i_ is the number of different values of X_i_’s parents, r_i_ is the number of values of X_i_. The score calculates the probability of observing the network given the data.
4. Select the Bayesian Networks that have the highest fitness, which will then undergo crossover and mutations. During crossover and mutation, parts of the different Bayesian Networks are exchanged or mutated to create new networks.
5. Repeat 3–4 for a set number of generations
6. Save the best model in the final generation and evaluate it on testing data

The final Bayesian Network is composed of connected and unconnected variables. Variables that are connected in the network are directly dependent with each other, while unconnected variables are conditionally independent. The advantage of GEBN over the more traditional network construction is that it can explore a wider search space, thus more suitable for large-scale genomics data. In addition, using an evolutionary search strategy removes the dependency on human trial and error to create optimal network structures and instead relies on the data and computation along with evolutionary learning to find optimal structures.

### Discriminant analysis

The above GEBN method is applied to the case group and the control group independently. To prevent over-fitting, we used Bayesian Information Criteria (BIC) [23] to control the model complexity. The BIC is calculated as:

$$ BIC=-2* \ln (L)+k* \ln (n) $$

Where L is the maximum likelihood of data given a network, k is the number of free parameters, and n is the sample size. We iteratively removed each edge in the case or control network and calculated BIC for the reduced model. If the reduced model had higher BIC value, the edge was retained, and vice versa.

Finally, we used the discriminant analysis to assign an individual into either the case group or the control group. Using Bayes theorem, the probability of the sample belonging to a case group is calculated by:

$$ P\left(Y= Case\Big| Data\right)=\frac{P\left( Data\Big|Y= Case\right)*P\left(Y= Case\right)}{P\left( Data\Big|Y= Case\right)*P\left(Y= Case\right)+P\left( Data\Big|Y= Control\right)*P\left(Y= Control\right)} $$

Where $P\left(Y= Case\right)$ and $P\left(Y= Control\right)$ are given by their proportions in the total sample and $P\left( Data\Big|Y= Case\right)$ is calculated as:

$$ P\left( Data\Big|Y= Case\right)=P\left( Data\Big|Y= Case, Case\ Net\right)={\displaystyle \prod_i^p}P\left({G}_i\Big| Case\ Net\right) $$

*p* = total number of variables. $P\left( Data\Big|Y= Control\right)$ was calculated in the same fashion.

### Genetic data simulation

**Fig. 2:**
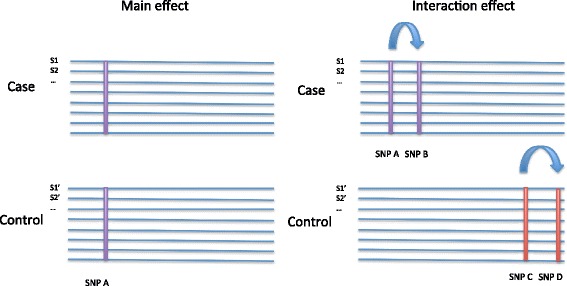
Schematic of data simulation. Main effect models have different allele frequencies in case and control datasets at the simulated SNPs. In interaction effect models, cases and control datasets have different simulated interacting SNPs without main effects

To test our approach, we simulated data that contains functional SNP variables with main effects and interaction effects. For main effect simulation, we simulated data that consist of different numbers of functional SNPs with varying degrees of association to a binary outcome. For interaction effects, we separately simulated a number of interaction effects in case and control groups. We purposely made the interaction effects different in case and control groups to mimic different genetic architectures in two groups (Fig. 2).

To simulate different degrees of main effect, for a functional SNP, we altered the allele frequencies in the case data (F_case_) using a weighted average of allele frequencies in the control data (F_control_) and the extreme allele frequencies (F_effect_) that were defined as (AA = 100 %, Aa = 0 %, aa = 0 %). Thus, the allele frequencies of the functional SNP in the case data is obtained by F_case_ = w*F_effect_ + (1-w)*F_control_, where w is the weight index – larger w indicating more discrepancy between the case frequencies and control frequencies.

The interaction effects were simulated as follows: Let F_ind_ denotes the joint frequencies of a pair of uncorrelated SNPs, which is calculated as the product of marginal frequencies between SNPs. The correlation can be increased by relocating the frequencies from the off-diagonal to the diagonal in the frequency table, and an extreme case is that only the diagonal have non-zero frequencies, which is denoted by F_diag_. Different strength of interactions can be simulated by w*F_diag_ + (1-w)* F_ind_.

For each dataset, we used the simulated frequency tables with sampling with replacement to determine the genotype of the functional SNPs. Then, we embedded the functional SNPs into a dataset with random SNPs to make it comparable to real biological datasets. Details of simulation parameters are shown in Table 1.

**Table 1:** Data simulation details

	Functional SNPs in case data	Functional SNPs in control data	Weight (W)	No. datasets for each W	Total SNPs	Sample size
Main effect	SNP A	SNP A	0.1, 0.5, 0.9	10	100, 500	4000
	SNP A, B, C, D	SNP A, B, C, D	0.1, 0.5, 0.9	10	100, 500	4000
Interaction effect	SNP A * SNP B	None	0.1, 0.5, 0.9	10	100, 500	4000
	SNP A * SNP B	SNP W * SNP X	0.1, 0.5, 0.9	10	100, 500	4000
	SNP C * SNP D	SNP Y * SNP Z				

### Marshfield PMRP type 2 diabetes dataset

The Marshfield PMRP is a biobank that has collected ~20,000 adult subjects’ biological samples and electronic health records [15]. We obtained SNPs data of type 2 diabetes cases and controls who were genotyped on Illumina Human660W-Quad BeadChip. We only retained individuals who are European Americans because they account for over 95 % of samples and we also removed related samples. For SNP quality control (QC), we kept SNPs that have 100 % call rate and minor allele frequency > 5 %. The cleaned data consists of 267, 209 SNPs in 800 cases and 2465 controls. We then performed a GWAS using logistic regression to identify a set of candidate SNPs with main effects for GEBN analysis (this is a main effects filtering step [24]). Association analysis was performed while adjusting for sex, median BMI, and birth decade. Case–control status for T2D was determined using Mount Sinai’s diabetes algorithm [25] from the Diabetes HTN CKD algorithm [26].

## Results and discussion

### Simulation results

In the simulation study, we compared the performance of GEBN to that of the traditional GWAS approach based on logistic regression and another widely used method for detecting interactions, grammatical evolution neural network (GENN) [27, 28]. The prediction performance is summarized by the respective receiver operating characteristic (ROC) curves and the area under the curve (AUC). For each setting, we show the prediction performance averaged over 10 simulations. Regression models that include the exact simulated model (MAX) are also used to show the upper bound of prediction performance.

**Fig. 3:**
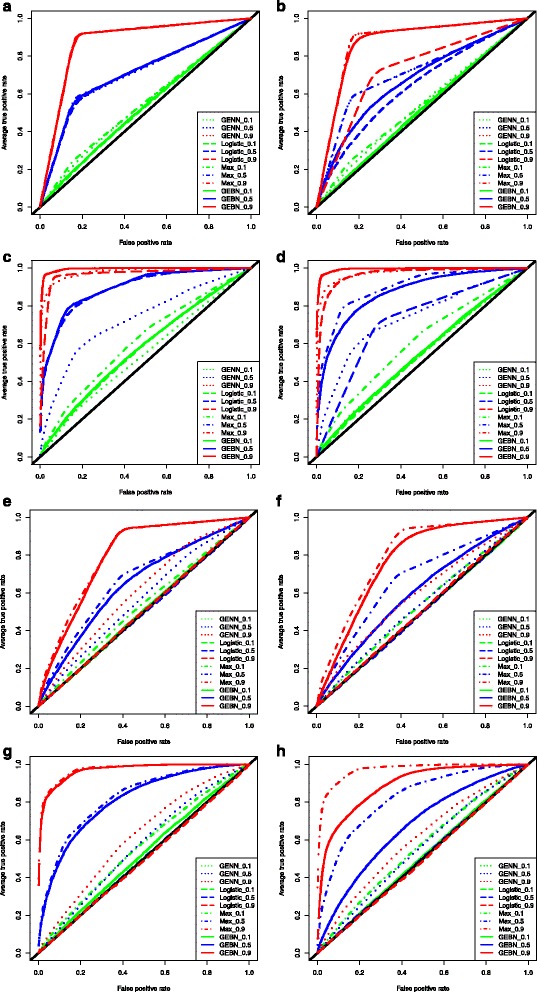
Simulation results for additive and interaction models using grammatical evolution Bayesian Network (GEBN), grammatical evolution neural network (GENN), logistic regression, and logistic regression with the exact simulated model (MAX). The colors represent different weight indexes (*red* = 0.9, *blue* = 0.5, *green* = 0.1). These weight indices correspond to strength of the simulated effects. **a**. Main effect model: SNP A (100) **b**. Main effect model: SNP A (500) **c**. Main effect model: SNP A, B, C, D (100) **d**. Main effect model: SNP A, B, C, D (500) **e**. Interaction model: SNP A < − > B (100) **f**. Interaction: SNP A < − > B (500) **g**. Interaction model: SNP A < − > B, C < − > D, W < − > X, Y < − > Z (100) **h**. Interaction model: SNP A < − > B, C < − > D, W < − > X, Y < − > Z (500)

For main effect models, GEBN achieved close to maximum prediction performance in datasets with 100 SNPs. Logistic regression showed similar power, while GENN showed lower power. With 500 SNPs, the performance advantage of GEBN is even more visible (Fig. 3a-d). The performance of all methods were improved by increasing the number of functional SNPs and increasing the effect size.

When case data and control data only differ by SNP interactions, logistic regression failed to separate two types with ROC curve fluctuating along the 45° line which corresponds to random guesses. GENN showed some power in detect interactions. However, GEBN showed improved ROC especially when the effect size is large (Fig. 3e-h). The execution time for GEBN depends on the parameter settings. With the current settings of population size of 3000 and 300 generations of evolution, the average running time is 1.5 ± 0.07 h for 100 SNPs and 0.97 ± 0.1 h for 500 SNPs and the running time is not dependent on the underlying model. The average AUC for all the models are listed in Table 2.

**Table 2:** Comparison of AUC for GEBN and logistic regression

	Functional SNPs in Case data	Functional SNPs in Control data	Weight (W)	MAX	Regression		GENN		GEBN	
				100	100	500	100	500	100	500
Main effect	SNP A	SNP A	0.1	55	52	51	54	54	53	52
			0.5	71	71	64	70	71	71	67
			0.9	88	88	72	88	88	88	87
	SNP A, B, C, D	SNP A, B, C, D	0.1	61	57	53	54	54	58	54
			0.5	90	89	72	73	73	89	87
			0.9	99	96	87	98	98	99	99
Interaction effect	SNP A * SNP B	None	0.1	53	50	50	50	50	50	50
			0.5	67	50	49	56	53	65	60
			0.9	89	50	50	60	59	80	77
	SNP A * SNP B	SNP W * SNP X	0.1	56	50	50	50	50	52	51
	SNP C * SNP D	SNP Y * SNP Z	0.5	82	50	50	57	55	81	67
			0.9	97	49	50	62	60	97	89

### Type 2 diabetes results

**Fig. 4:**
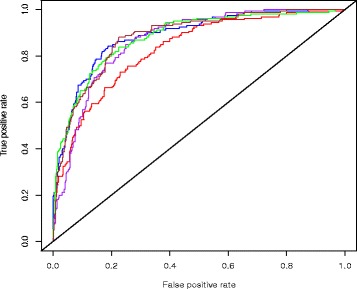
Testing ROC curve for type 2 diabetes. Each color represents a single cross-validation

We first performed association analysis using logistic regression for 267,209 SNPs associations with type 2 diabetes, using *p* < 0.001 as threshold, we identified 259 SNPs associated with type 2 diabetes. The top associated SNP was rs7903146 (*p* = 2.997e–06), which maps to *TCF7L2* gene. To remove SNPs that are correlated, we used PLINK software [29] to prune the associated SNPs based on linkage disequilibrium (−−indep 50 5 2). 202 SNPs remained after LD pruning. We applied GEBN on the 202 SNPs, together with sex, median BMI, and birth decade, to separately build interaction networks for type 2 diabetes cases and controls. We then used the final network from cases and controls to perform discriminate analysis on the independent testing data. The average prediction AUC of 5-fold cross validation was 86.8 % (Fig. 4).

**Fig. 5:**
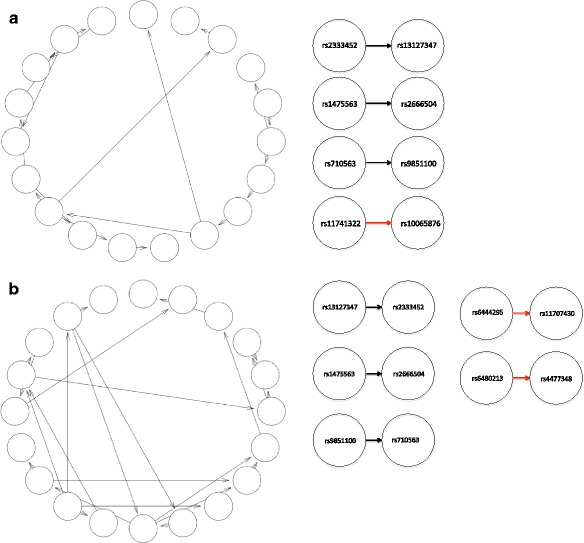
Best Bayesian Network models for cases and controls. Left panel shows network structure before BIC pruning. Right panel shows network structure after BIC pruning, and the red edges indicate interactions only found in the case data or the control data, but not both cases and controls. **a**. Case data network, **b**. Control data network

Figure 5 shows the best Bayesian Network models for cases and controls. The AUC for the best model was 88.7 %. The networks also include the rest of the SNPs as marginal variables, but for clarity, they were not shown. The cases and controls share there common interactions: rs13127347 and rs2333452, rs9851100 and rs710563 (both in *P3H2* gene), rs2666504 and rs1475563 (*INADL* gene). There was also one unique interaction for cases, which is rs10065876 and rs11741322 and two for controls, which are rs4477348 and rs6480213 (both in *CTNNA3* gene), and rs11707430 and rs6444295 (both in *LPP* gene).

## Conclusions

In this study, we presented a novel algorithm that can efficiently capture marginal and interaction effects present in the genetic data. We demonstrated in simulation data that GEBN performed equal or better than the standard GWAS analysis method using logistic regression as well as GENN on data with only main effect functional SNPs. In data with interacting SNPs, logistic regression failed to capture the true model which is shown by the ~50 % AUC (Fig. 2). GENN was able to capture simulated interactions, however, the predictive power were significantly lower than the MAX models, which gives the upper bound of prediction performance. On the other hand, GEBN were able to separately identify the unique interactions in cases and controls and use that information to distinguish the two groups. The performance of GEBN was close to the maximum prediction power in data with 100 and 500 SNPs. One concern was that GEBN can potentially over fit the data because networks were trained separately for each group. However, our testing AUCs showed that we did not over fit the model.

Using main effect filtering followed by GEBN analysis, we replicated canonical associations and also identified novel genetic interactions for type 2 diabetes. The most significant association was rs7903146, which is located in the *TCF7L2* gene. We also identified rs12255372, which is in LD with rs7903146, as a significant association. *TCF7L2* gene has been implicated for type 2 diabetes in many studies [30, 31]. We limited the network analysis to the top 202 associated SNPs because it is a comparable size to our simulation study. It is interesting that the top case and control networks have common as well as unique edges. The common edges include two non-coding SNPs on chromosome 4, two SNPs within *P3H2* gene and one SNP in *INADL* gene and one SNP in the non-coding region of chromosome 1. The *INADL* gene is part of the hippo signaling pathway [32]. The pathway has been shown to regulate pancreas development [33] and adipocyte development [34]. Interestingly, a prior study has found that *INADL* was associated with children’s weight [35]. It is difficult to interpret the unique interaction for case group because both of the SNPs are located in non-coding regions. These could be further analyzed by looking into the ENCODE and GTEx regulatory data for possible functions. For controls, *CTNNA3* were found to be associated with Alzheimer [36] and heart disease [37]. *LPP* gene has shown a robust association with type 2 diabetes in multiple ethnicities as well as combined meta-analysis [38]. Taken together, we have shown that GEBN have identified several known genes associated with type 2 diabetes. Using logistic regression, we also obtained a similar prediction AUC of 86.5 %. The similar performance was mostly due to the candidate SNPs were selected using a main effect filtering. Despite the similarity in the AUCs, GEBN was able to identify more complex genetic structures in diabetes cases and controls than logistic regression.

This paper presents the first step of the algorithm development that aims to address the pressing need for tools to identify complex relationships within the genetics data. Due to the flexibility of the Bayesian networks, the algorithm could be applied to datasets with more than two outcomes. For example, drug response phenotypes might be categorized as high responder, low responder, and non-responder. This would be possible to analyze with GEBN.

The utility of GEBN will be even greater in those settings because traditional statistical approaches are generally limited to binary outcomes. We also plan to integrate other –omics data such as transcriptomic and methylomic data into the network. The potential interactions between factors from different data types could reveal novel biological insights not seen at any individual data alone. The ultimate goal of individually identifying networks for different groups or subtypes of disease is to more precisely understand the disease so that we can improve detection and treatment of the disease. The method presented in this paper will help further elucidate the complex biological relationship present in the genetics data.
